# CRISPR/Cas9-mediated targeted mutagenesis of *GmSPL9* genes alters plant architecture in soybean

**DOI:** 10.1186/s12870-019-1746-6

**Published:** 2019-04-08

**Authors:** Aili Bao, Haifeng Chen, Limiao Chen, Shuilian Chen, Qingnan Hao, Wei Guo, Dezhen Qiu, Zhihui Shan, Zhonglu Yang, Songli Yuan, Chanjuan Zhang, Xiaojuan Zhang, Baohui Liu, Fanjiang Kong, Xia Li, Xinan Zhou, Lam-Son Phan Tran, Dong Cao

**Affiliations:** 1 Key Laboratory of Biology and Genetic Improvement of Oil Crops, Ministry of Agriculture, Oil Crops Research Institute, Chinese Academy of Agricultural Sciences, Wuhan, 430062 China; 2 The Key Laboratory of Soybean Molecular Design Breeding, Northeast Institute of Geography and Agroecology, Chinese Academy of Sciences, Harbin, 150081 China; 3 State Key Laboratory of Agricultural Microbiology, College of Plant Science and Technology, Huazhong Agricultural University, Wuhan, 430070 China; 4 Institute of Research and Development, Duy Tan University, 03 Quang Trung, Da Nang, Vietnam; 5 Stress Adaptation Research Unit, RIKEN Center for Sustainable Resource Science, 1-7-22, Suehiro-cho, Tsurumi, Yokohama, 230-0045 Japan

**Keywords:** CRISPR/Cas9, Plastochron length, Plant architecture, Soybean, *SPL*

## Abstract

**Background:**

The plant architecture has significant effects on grain yield of various crops, including soybean (*Glycine max*), but the knowledge on optimization of plant architecture in order to increase yield potential is still limited. Recently, CRISPR/Cas9 system has revolutionized genome editing, and has been widely utilized to edit the genomes of a diverse range of crop plants.

**Results:**

In the present study, we employed the CRISPR/Cas9 system to mutate four genes encoding SQUAMOSA PROMOTER BINDING PROTEIN-LIKE (SPL) transcription factors of the SPL9 family in soybean. These four *GmSPL9* genes are negatively regulated by *GmmiR156b*, a target for the improvement of soybean plant architecture and yields. The soybean Williams 82 was transformed with the binary CRISPR/Cas9 plasmid, assembled with four sgRNA expression cassettes driven by the *Arabidopsis thaliana* U3 or U6 promoter, targeting different sites of these four *SPL9* genes via *Agrobacterium tumefaciens*-mediated transformation. A 1-bp deletion was detected in one target site of the *GmSPL9a* and one target site of the *GmSPL9b*, respectively, by DNA sequencing analysis of two T0-generation plants. T2-generation *spl9a* and *spl9b* homozygous single mutants exhibited no obvious phenotype changes; but the T2 double homozygous mutant *spl9a*/*spl9b* possessed shorter plastochron length. In T4 generation, higher-order mutant plants carrying various combinations of mutations showed increased node number on the main stem and branch number, consequently increased total node number per plants at different levels. In addition, the expression levels of the examined *GmSPL9* genes were higher in the *spl9b-1* single mutant than wild-type plants, which might suggest a feedback regulation on the expression of the investigated *GmSPL9* genes in soybean.

**Conclusions:**

Our results showed that CRISPR/Cas9-mediated targeted mutagenesis of four *GmSPL9* genes in different combinations altered plant architecture in soybean. The findings demonstrated that GmSPL9a, GmSPL9b, GmSPL9c and GmSPL9 function as redundant transcription factors in regulating plant architecture in soybean.

**Electronic supplementary material:**

The online version of this article (10.1186/s12870-019-1746-6) contains supplementary material, which is available to authorized users.

## Background

Soybean (*Glycine max*) plant architecture is an important trait for developing high-yield cultivars, and this trait can be determined based on stem growth habit, node number, plant height, internode length, branch number, leaf size and shape [1, 2]. Previous studies on soybean plant architecture have primarily focused on stem growth habit [1, 3–7]. Recently, Gao et al. (2017) found that the *Glycine max INCREASED LEAF PETIOLE ANGLE 1* (*GmILPA1*), a gene encoding an ‘anaphase-promoting complex/cyclosome’ (APC/C) protein, modulated the leaf petiole angle in soybean [8]. In addition, marker-assisted studies have revealed many quantitative trait loci (QTLs) associated with various traits related to plant architecture in soybean, including plant height, internode length, node number, branch number, pod number, and leaflet length and width (http://www.SoyBase.org). However, the molecular mechanisms regulating plant architecture and yield potential remain unknown, and information about the genes responsible for improving soybean plant architecture is still limited.

In plants, most members of the *SQUAMOSA PROMOTER BINDING PROTEIN-LIKE* (*SPL*) transcription factor (TF) family are regulated through *miR156*, and these TFs affect the transition between the juvenile and adult phases [9–12]. In *Arabidopsis*, *SPL9* and *SPL15* have been shown to be implicated in the regulation of plastochron length and leaf size [13, 14]. In rice (*Oryza sativa*), *OsSPL14* has been identified as *IDEAL PLANT ARCHITECTURE 1* (*IPA1*) or *WEALTHY FARMER’S PANICLE* (*WFP*) gene, which regulates shoot branching during the vegetative phase and the number of grains produced in a panicle [15, 16]. The rice *OsSPL14* gene encodes the closest homologous protein of the *Arabidopsis* SPL9 and SPL15, and its overexpression also prolongs plastochron length [17]. The OsSPL14 can directly bind to the promoter of the *TEOSINTE BRANCHED 1* (*TB1*) in rice to suppress rice tillering, and positively and directly regulates the expression of *DENSE AND ERECT PANICLE 1* (*DEP1*) to affect plant height and panicle length [18]. Wang et al. (2015) reported that a spatiotemporally coordinated gene network comprising the *miR156*/*miR529*/*SPL* and *miR172*/*Apetala2* (*AP2*) pathways controls tiller and panicle branching in rice [19]. Recently, Wang et al. (2017) identified a RING-finger E3 ligase, named IPA1 INTERACTING PROTEIN 1 (IPI1), that can interact with OsSPL14 in the nucleus [20]. IPI1 promotes the degradation of OsSPL14 in panicles, while it stabilizes OsSPL14 in shoot apexes, thereby regulating plant architecture in rice [20]. In soybean, transgenic plants overexpressing the *GmmiR156b* produced greatly altered plant architecture, leading to a remarkable increase in grain yield per plant [21]. It has also been reported in soybean that the *GmSPL9d* gene is expressed in the shoot apical meristem (SAM) and axillary meristem (AM), and that GmSPL9d may regulate axillary bud formation and shoot branching by physically interacting with the homeobox protein WUSCHEL (WUS), a central regulator of AM formation [21]. *GmmiR156b* regulates soybean plant architecture mainly through the direct cleavage of *SPL* genes [21]. However, our knowledge on the functions of *GmSPL9* genes in controlling plant architecture is still limited in soybean.

Recently, the emergence of clustered regularly interspaced short palindromic repeats/CRISPR associated protein 9 (CRISPR/Cas9) technology has brought new opportunities to the field of genetic manipulation in plants [22–24]. It has attracted large attention, and its application has dramatically expanded in genome editing of many crops, including rice [7, 23], wheat (*Triticum aestivum*) [25–27], maize (*Zea mays*) [28, 29], oilseed rape (*Brassica napus*) [30], barley (*Hordeum vulgare*) [31], cotton (*Gossypium hirsutum*) [32], tomato (*Solanum lycopersicum*) [33] and soybean [34–36]. Very recently, Cai et al. (2018) have reported the successful application of the CRISPR/Cas9 system in soybean in mutating the gene *Flower Locus T* (*FT*), which resulted in delayed flowering time of mutated plants under both short-day and long-day conditions, suggesting that gene knock-out mediated by the CRISPR/Cas9 system in soybean research is feasible [36]. However, research in soybean using the CRISPR/Cas9 system is still rare, due to the fact that soybean transformation is still a great challenge for most research groups. Furthermore, most of the targets of the successful applications of the CRISPR/Cas9 system in gene editing in soybean were single gene [34–36]. Here, we report the CRISPR/Cas9-based multiple gene editing system to target four *SPL9* genes in soybean. T4-generation soybean mutant plants carrying different combinations of mutations exhibited a number of altered characteristics in plant architecture. Our findings indicate that the CRISPR/Cas9 system is a promising tool to advance soybean breeding.

## Results

### Target selection and construction of the CRISPR/Cas9 vector system for mutagenesis of four *GmSPL* genes in soybean

**Fig. 1:**
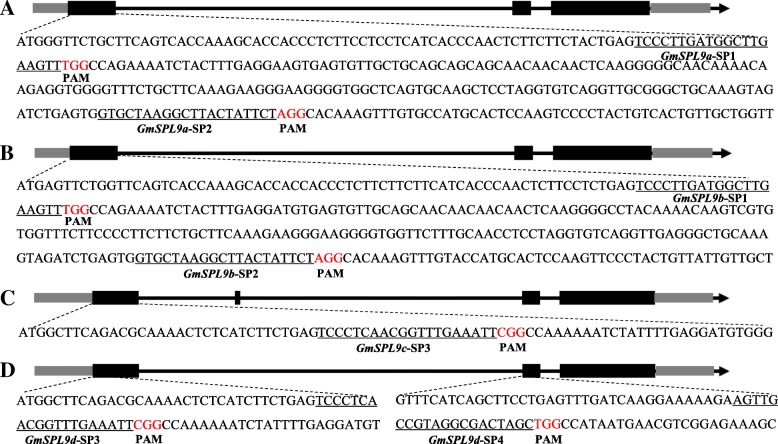
Schematic figure of target sites in four *GmSPL9* genes. (**a**) Gene structure of *GmSPL9a* with two target sites *GmSPL9a*-SP1 and *GmSPL9a*-SP2. (**b**) Gene structure of *GmSPL9b* with two target sites *GmSPL9b*-SP1 and *GmSPL9b*-SP2. (**c**) Gene structure of *GmSPL9c* with one target site *GmSPL9c*-SP3. (**d**) Gene structure of *GmSPL9d* with two target sites *GmSPL9d*-SP3 and *GmSPL9d*-SP4. Nucleotides in red represent the protospacer adjacent motif (PAM). Nucleotides underlined indicate the target sites. Gray stripe, untranslated regions; black stripe, exon; black line, intron

**Fig. 2:**
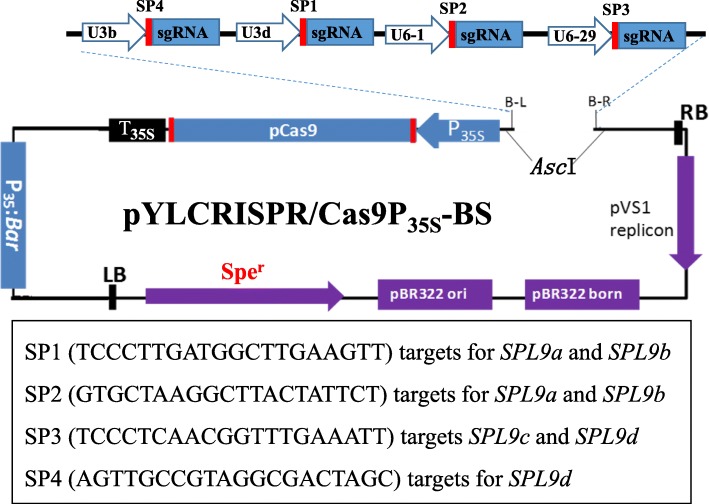
Schematic figure of the binary vector designed for mutagenesis of the four *GmSPL9* genes using the CRISPR/Cas9 technology. The pYLCRISPR/Cas9P_35S_-BS was derived from the pYLCRISPR/Cas9P_35S_-B [38]. The target adaptor SP1, targeting two sites (*GmSPL9a*-SP1 and *GmSPL9b*-SP1), directed by the *Arabidopsis thaliana* U3d promoter; the target adaptor SP2, targeting two sites (*GmSPL9a*-SP2 and *GmSPL9b*-SP2), directed by the *A. thaliana* U6–1 promoter; the target adaptor SP3, targeting two sites (*GmSPL9c*-SP3 and *GmSPL9d*-SP3), directed by the *A. thaliana* U6–29 promoter; the target adaptor SP4, targeting one site (*GmSPL9d*-SP4), directed by the *A. thaliana* U3b promoter

It has been reported that *GmmiR156b* overexpression improved yield-related phenotypic traits in soybean [21], suggesting the involvement of the *GmSPL* genes, which are the cleavage targets of *GmmiR156b* [21], in regulating the architecture of soybean plants in a negative manner. This hint was strengthened by the fact that the *GmSPL9a*, *GmSPL9b*, *GmSPL9c* and *GmSPL9d* genes were down-regulated in *GmmiR156b*-overexpressing transgenic soybean plants [21, 37]. Additional file 1: Figure S1 showed that the GmSPL9a, GmSPL9b, GmSPL9c and GmSPL9d were clustered into the AtSPL9/AtSPL15 and OsSPL14/OsSPL17 cluster, suggesting that all four GmSPL9 TFs might have a role in altering the architecture of soybean plants. To study their function by a genetic means, three target adaptors, SP1 (selected for targeting *GmSPL9a* and *GmSPL9b* genes), SP2 (selected for targeting *GmSPL9a* and *GmSPL9b* genes) and SP3 (selected for targeting *GmSPL9c* and *GmSPL9d* genes) in the first exon of these four genes, and one target adaptor (SP4) in the second exon of *GmSPL9d* were chosen for mutagenesis of these four genes in soybean using the CRISPR/Cas9 technology (Fig. 1). The *Arabidopsis* U3b, U3d, U6–1 and U6–29 promoters were used to drive the individual expression of the 4 sgRNA expression cassettes containing the designed target sites (Fig. 2). These constructs were inserted into the CRISPR/Cas9 vector system designed previously [38] (Fig. 2), and the obtained plasmid was introduced into the soybean Williams 82 variety using *Agrobacterium tumefaciens* according to the procedure described by Cao et al., 2015 [37].

### Targeted mutagenesis of four *GmSPL9* genes in soybean

**Fig. 3:**
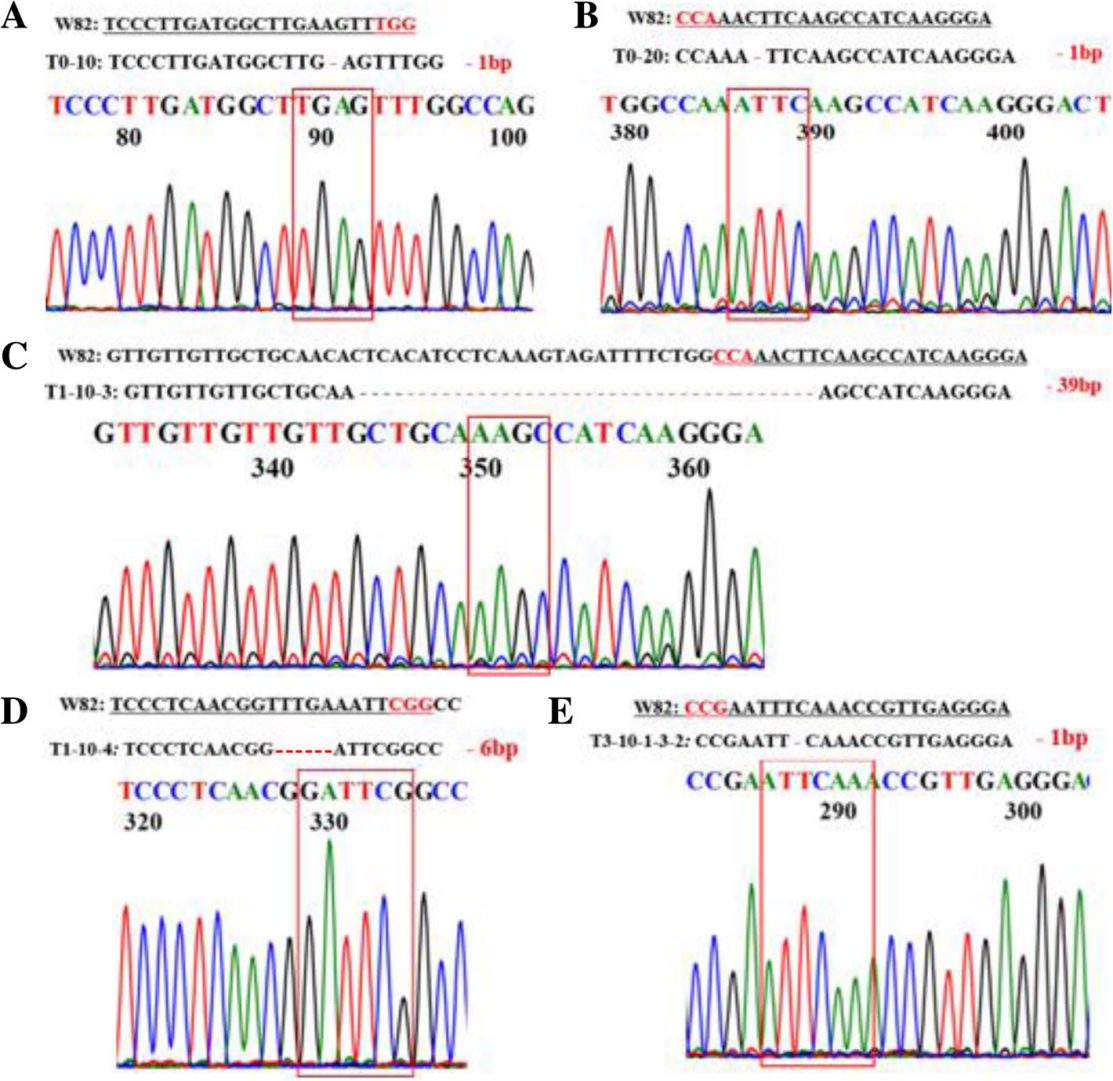
Results obtained from mutagenesis of four *GmSPL9* genes by CRISPR/Cas9 technology. (**a**) Detailed sequence of the target site *GmSPL9a*-SP1 in the T0–10 line. (**b**) Detailed sequence of the target site *GmSPL9b*-SP1 in the T0–20 line. (**c**) Detailed sequence of the target site *GmSPL9b*-SP1 in the T1–10-3 line. (**d**) Detailed sequence of the target site *GmSPL9c*-SP3 in the T1–10-4 line. (**e**) Detailed sequence of the target site *GmSPL9d*-SP1 in the T3–10–1-3-2 line. Nucleotides in red and underlined represent the protospacer adjacent motif (PAM). The underlined nucleotides indicated the target sites. ‘-’ signs indicate the number of deleted nucleotides. W82 represents Williams 82 wild-type sequence

We obtained two T0 transgenic lines with the section for the *Bar* gene product (*Bar*-positive). Genomic DNA was extracted from leaves using cetyltrimethyl ammonium bromide (CTAB) to investigate CRISPR/Cas9-induced mutations at the target sites. Sequencing analysis showed that the T0–10 line had a 1-bp deletion in the *GmSPL9a*-SP1 (Fig. 3a, *spl9a* allele; Additional file 2: Table S1), while the T0–20 line had a 1-bp deletion in the *GmSPL9b*-SP1 (Fig. 3b, *spl9b-1* allele; Additional file 2: Table S1; Additional file 3: Figure S2A), resulting in frame-shift mutations in both *GmSPL9a* and *GmSPL9b* genes (Additional file 2: Table S1; Additional file 3: Figure S2A). Both these two mutations generated premature translation termination codons (PTCs), and thus are null mutations (Additional file 3: Figure S2A; Additional file 4: Text S1). However, we found that the other five target sites *GmSPL9a*-SP2, *GmSPL9b*-SP2, *GmSPL9c*-SP3, *GmSPL9d*-SP3 and *GmSPL9d*-SP4 showed no edited mutations in both two T0 plants. Subsequently, we analyzed four T1–10 plants and six T1–20 plants and found two new edited types; one in the target site *GmSPL9b*-SP1 (39-bp deletion) (Fig. 3c, *spl9b-2* allele; Additional file 2: Table S1; Additional file 3: Figure S2A) and another in the target site *GmSPL9c*-SP3 (6-bp deletion) (Fig. 3d, *spl9c* allele; Additional file 2: Table S1; Additional file 3: Figure S2B). The 39-bp deletion resulted in a 12-amino-acid deletion (from position 28 to 39) and an amino-acid substitution (F40 V) in the GmSPL9b protein (Additional file 3: Figure S2A), while the 6-bp deletion caused a deletion of two amino acids in the GmSPL9c protein (from position 16 to 17) (Additional file 3: Figure S2B).

There is currently not much knowledge with respect to the functions of *GmSPL9a*, *GmSPL9b* and *GmSPL9c* on the regulation of plant architecture. However, there was a report about the function of *GmSPL9d* with regard to its regulatory function in plant architecture. Specifically, overexpression of the *GmSPL9d* gene suppressed the branch number in *Arabidopsis* transgenic plants [21]. Thus, given the redundant functions of the *GmSPL9* genes, to obtain profound evidence for their genetic involvement in regulating plant architecture, we were interested in identifying the higher-order mutants, especially those that contain mutation in the *GmSPL9d* gene (Fig. 3e; Additional file 2: Table S1). The seeds of ten T1-generation plants (four T1–10 plants and six T1–20 plants) were sown, and the DNAs of 120 independent T2 plants (12 independent T2 plants from each T1-generation plant) were obtained. We then mixed the DNAs of 12 independent T2 plants from each T1-generation plant as one pooled DNA template for PCR, resulting in 10 DNA pools. Sequence analysis showed that there were no edited mutations in the two target sites of *GmSPL9d* (*GmSPL9d*-SP3 and *GmSPL9d*-SP4) among the examined T2 plants. When we obtained the T3-generation seeds, we conducted similar experiments to identify *spl9d* mutants. The pooled DNAs of T3–10–1-3 (mixed 12 plants) and T3–10–1-6 (mixed 12 plants) had a new edited type in *GmSPL9d* (*spl9d* allele, Additional file 2: Table S1). Further sequence analysis showed a 1-bp deletion in the target site of *GmSPL9d-SP3* in T3–10–1-3-2 (Fig. 3e; Additional file 2: Table S1), resulting in the *spl9a* (−/−)/*spl9b-1* (−/−)/*spl9d* (+/−) mutant. In addition, we also obtained a ‘transgene-clean’ *spl9b-1* (−/−) mutant line from the T2–10–1-1 line using the selectable marker gene *Bar* for selection (Additional file 2: Table S1; Additional file 5: Figure S3). The T2–10–1-1 line had 1-bp deletion in the target site of *GmSPL9b*-SP1, and its T3 and T4-generation plants were all ‘transgene-clean’ homozygous *spl9b-1* mutants. After four generations of selection, we obtained the ‘transgene-clean’ homozygous *spl9b-1* single and the *spl9a*/*spl9b-1*/*spl9c*/*spl9d* homozygous quadruple mutants, and some other mutants that were still *Bar*-positive like *spl9a* (−/−)/*spl9b-1* (−/−), *spl9a* (−/−)/*spl9b-1* (−/−)/*spl9c* (+/−), *spl9a* (−/−)/*spl9b-2* (−/−)/*spl9c* (+/−) and *spl9a* (−/−)/*spl9b-1* (−/−)/*spl9c* (+/−)/*spl9d* (+/−) from our genome editing experiment (Additional file 2: Table S1).

### The examined four *SPL9* genes regulate soybean plant architecture

**Fig. 4:**
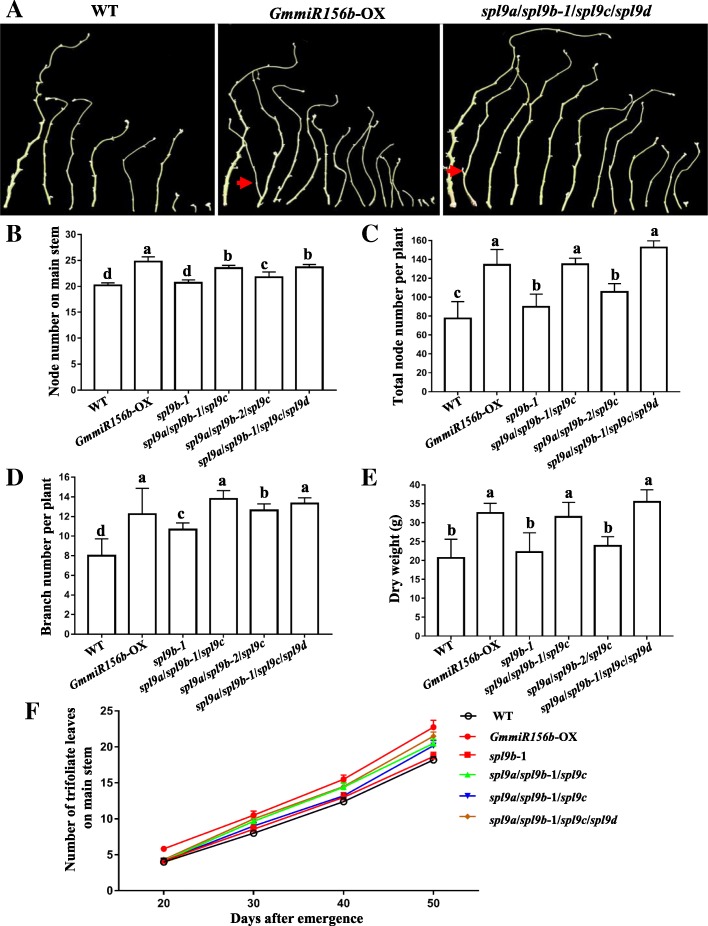
The soybean CRISPR/Cas9-induced T4-generation *spl9* mutant lines showed altered plant architecture. (**a**) Representative pictures showing the primary and secondary branches of wild-type (WT), *GmmiR156b*-overexpressing (*GmmiR156b-*OX) and *spl9a* (−/−)/*spl9b-1* (−/−)/*spl9c* (−/−)/*spl9d* (−/−) quadruple mutant plants at 50th day after emergence, after the leaves were removed. Red arrows indicate the secondary branches on primary branches. (**b**) Node number on main stem, (**c**) total node number per plant, (**d**) branch number, and (**e**) dry weight were recorded after the experiment was completed at day 50th after emergence. (**f**) Dynamic changes in the trifoliate leaf number on main stem in different genotypes. (**b**-**f**) The data of WT, *GmmiR156b-*OX and the *spl9b-1* (−/−) single mutant plants were obtained from six plants. The data of *spl9a*/*spl9b-1*/*spl9c* were obtained from five *spl9a* (−/−)/*spl9b-1* (−/−)/*spl9c* (+/−) plants, the data of *spl9a*/*spl9b-2*/*spl9c* were obtained from nine *spl9a* (−/−)/*spl9b-2* (−/−)/*spl9c* (+/−) plants, and the data of *spl9a*/*spl9b-1*/*spl9c*/*spl9d* were obtained from two *spl9a* (−/−)/*spl9b-1* (−/−)/*spl9c* (−/−)/*spl9d* (−/−) and one *spl9a* (−/−)/*spl9b-1* (−/−)/*spl9c* (+/−)/*spl9d* (+/−) plants. “a, b, c and d” indicate statistically significant differences among the genotypes (one-way ANOVA of variance, *P* < 0.05)

In the T1 and T2 generations, the obtained single mutants *spl9b-1* and *spl9c* showed no differences in node number on main stem as compared with wild-type (WT) plants, whereas the obtained *spl9a*/*spl9b-1* double mutant plants had one more trifoliate leaf than the WT when they were grown in an artificial climate chamber at 12-h light/12-h dark photoperiod and 24 °C (Additional file 6: Figure S4a). When we were growing the different T4 mutant plants under artificial climate chamber at 15-h light/9-h dark photoperiod and 28 °C, we found that they exhibited notable changes in plant architecture (Fig. 4). For instance, similar to transgenic plants overexpressing *GmmiR156b* (*GmmiR156b-*OX), the T4 *spl9a*/*spl9b-1*/*spl9c*/*spl9d* homozygous quadruple mutant plants showed more branches than WT, including some secondary branches originated from the primary branches (Fig. 4a). The T4 ‘transgene-clean’ homozygous *spl9b-1* single mutant plants showed no difference in node number on main stem (Fig. 4b), which was in agreement with the results obtained in the T1- and T2-generation *spl9b-1* (−/−) mutant plants, but the *spl9b-1* single mutant plants exhibited 15.5 and 33.0% increase in total node number per plant and branch number, respectively, as compared with that of WT plants (Fig. 4c-d). Furthermore, the T4 *spl9a* (−/−)/*spl9b-1* (−/−)/*spl9c* (+/−) and *spl9a* (−/−)/*spl9b-2* (−/−)/*spl9c* (+/−) mutant plants showed 16.3 and 7.7% increase in node number on main stem, 73.7 and 36.3% increase in total node number per plant, 72.5 and 57.8% increase in branch number, and 52.2 and 15.2% increase in dry weight, respectively, relative to that of WT plants (Fig. 4b-e). Interestingly, the T4 *spl9a* (−/−)/*spl9b-1* (−/−)/*spl9c* (+/−) mutant plants exhibited more remarkable phenotypic changes in the parameters examined compared with the T4 *spl9a* (−/−)/*spl9b-2* (−/−)/*spl9c* (+/−) mutant plants (Fig. 4b-e), suggesting the mutation in *spl9b-1* was more severe than that in *spl9b-2*. Additionally, the T4 *spl9a* (−/−)/*spl9b-1* (−/−)/*spl9c* (+/−)/*spl9d* (+/−) and *spl9a* (−/−)/*spl9b-1* (−/−)/*spl9c* (−/−)/*spl9d* (−/−) mutant plants, when analyzed together, generally displayed the most significant changes in plant architecture when comparing with WT and the lower-order mutants, showing the highest node number on main stem, branch number, total node number per plant and dry weight among the examined genotypes (Fig. 4b-e). It is worth noting that these highest-order mutant plants, when analyzed together, showed a 13.2% increase in total node number per plant and a 12.6% increase in dry weight (Fig. 4c and e), but similar node number on main stem and branch number (Fig. 4b and d), in comparison with the T4 *spl9a* (−/−)/*spl9b-1* (−/−)/*spl9c* (+/−) mutant plants. Taken together, our data indicated that all four *SPL9* genes have important roles in regulating soybean plant architecture, both redundantly and independently.

### The examined four *SPL9* genes regulate plastochron length in soybean

The T4-generation *spl9b-1* single homozygous mutant showed no difference in number of trifoliates compared with WT, whereas the other T4 higher-order mutant plants examined showed shorter plastochron lengths in soybean compared with the *spl9b-1* single mutant and WT plants (Fig. 4f). In particular, 50 days after emergence (DAE), WT, *GmmiR156b-*OX, *spl9b-1*, *spl9a* (−/−)/*spl9b-1* (−/−)/*spl9c* (+/−) and *spl9a* (−/−)/*spl9b-2* (−/−)/*spl9c* (+/−) exhibited trifoliate leaves on the main stem in an average number of 18.2, 22.8, 18.7, 20.5 and 20.2, respectively, while the *spl9a* (−/−)/*spl9b-1* (−/−)/*spl9c* (+/−)/*spl9d* (+/−) and *spl9a* (−/−)/*spl9b-1* (−/−)/*spl9c* (−/−)/*spl9d* (−/−) mutant plants, when analyzed together, had the average number of trifoliate leaves of 21.5 (Fig. 4f). In addition, both the T2-generation *spl9a* (−/−)/*spl9b-1* (−/−) double mutant and *GmmiR156b-*OX plants had shorter plastochron lengths compared with WT plants (Additional file 6: Figure S4). These data indicated that these four *GmSPL9* genes are implicated in regulation of plastochron length in soybean, perhaps under the control of *GmmiR156b*.

### GmSPL9b regulates expression of the four *GmSPL9* genes

**Fig. 5:**
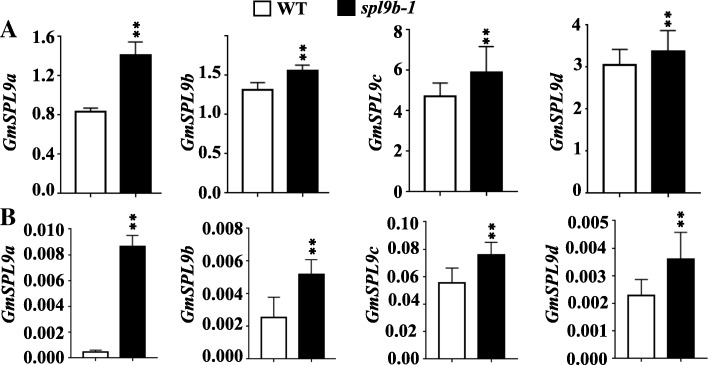
Expression patterns of four *GmSPL9* genes in wild-type (WT) and the *spl9b-1* (*Bar* negative) mutant plants. (**a**) Relative expression of *GmSPL9a*, *GmSPL9b*, *GmSPL9c* and *GmSPL9d* in shoot apical meristem of WT and *spl9b-1* plants. (B) Relative expression of *GmSPL9a*, *GmSPL9b*, *GmSPL9c* and *GmSPL9d* in leaves of WT and *spl9b-1* plants. The plants were grown under artificial climate chamber (15-h light/9-h dark) conditions for 20 days after emergence. Relative transcript levels were assessed by quantitative RT-PCR and normalization to the expression level of the *GmTUB* gene. Expression levels shown are means ± SEs of three replicates (**P < 0.01; Student’s *t*-test)

To validate the effect of the mutation in *GmSPL9b* gene (*spl9b-1* allele) on the expression of the four *GmSPL9* genes in soybean, we analyzed the expression levels of all four *GmSPL9* genes in leaves and SAM of the stable *spl9b-1* (*Bar* negative) single mutant and WT plants grown under artificial climate chamber (15-h light/9-h dark) at 20th DAE using quantitative RT-PCR (qRT-PCR). Figure 5 showed that the transcript levels of all four *GmSPL9* genes were higher in both SAM and leaves of the *spl9b-1* single mutant than in that of WT plants. To further validate whether the GmSPL9b TF regulates the expression of *GmSPL9a*, *GmSPL9c* and *GmSPL9d* genes in soybean, we generated transgenic lines overexpressing the *GmSPL9b*. The two independent transgenic lines displayed higher expression levels of the *GmSPL9b* gene in leaves than WT (Additional file 7: Figure S5A). Furthermore, our data revealed that the transgenic plants, especially line #5 with higher expression level of *GmSPL9b*, had lower transcript levels of *GmSPL9a*, *GmSPL9c* and *GmSPL9d* than WT plants (Additional file 7: Figure S5B-D). However, similar to the *spl9b-1* single mutant plants, the transgenic lines overexpressing *GmSPL9b* showed comparable plant architecture as WT plants as evidenced by the data of node number on main stem, total node number per plant and branch number per plant (Additional file 7: Figure S5E-G). Taken together, these results indicated that *GmSPL9b* gene may repress the expression of *GmSPL9a, GmSPL9c* and *GmSPL9d*, as well as itself in soybean.

## Discussion

### CRISPR/Cas9 genome editing efficiency in soybean - single construct for multiple mutations

In the current study, we explored the CRISPR/Cas9 system for mutagenesis of four *GmSPL9* genes by using a single plasmid construct in an attempt to make loss-of-function soybean mutants to assess the functions of these genes in regulation of plant architecture (Figs. 1-2). We designed four target adaptors (SP1, SP2, SP3 and SP4) and found that only the SP1 adaptor targeting *GmSPL9a* and *GmSPL9b* genes produced heterozygous mutants in T0-generation plants (Fig. 1; Additional file 2: Table S1). However, the editing continued to work with the constructed CRISPR/Cas9 system in plants of later generations (*Bar*-positive), and new mutants were obtained at different target sites (Additional file 2: Table S1). Our data indicated that the genome editing efficiency was low in T0-generation of soybean, but relatively high in T1-generation plants (Additional file 2: Table S1). Similar results were reported in *Arabidopsis*, which showed that the editing efficiency and editing types induced by CRISPR/Cas9 were relatively low, and both uniform and chimeric mutations were occurred in the T1-generation [38–40]. It has been suggested that *Arabidopsis* transformation methods using vegetative tissues might produce relatively low editing efficiency [38]. Thus, the low editing efficiency in the soybean T0-generation observed in this study might also be caused by our transformation method using cotyledonary node. Furthermore, we observed that the SP2 and SP4 adaptors designed for three target sites (*GmSPL9a* and *GmSPL9b* genes, and *GmSPL9d* gene, respectively) showed no edits in T0-, T1-, T2-, T3- and T4-generation plants. Previous studies reported that selection of target sequences with relatively higher GC content might result in a higher editing efficiency [38]. We should select target sites with higher GC content to improve the editing efficiency. Therefore, at least in the case of soybean, a crop with low transformation frequency [41], less target genes with more target sites in each target gene should be designed to make sure that lower-order mutants can be generated. Subsequently, higher-order mutants can be obtained through crossing.

### Functions of the four examined *GmSPL9* genes in regulating plant architecture of soybean

In rice, many studies have reported that the *OsSPL14* had a great role in regulation of plant architecture [15–20]. In *Arabidopsis*, SPL9, SPL15 and SPL10 function in a redundant manner to regulate plastochron length [13, 14]. In bread wheat, *miR156* was reported to control plant architecture via the repression of a group of *SPL* genes [42]. The SPL TFs share a highly conserved DNA-binding domain called SQUAMOSA PROMOTER BINDING PROTEIN (SBP)-box [42]. Several maize SBP*-*box-type TFs, such as TEOSINTE GLUME ARCHITECTURE (TGA1) [43], TASSELSHEATH4 (TSH4) [44], UNBRANCHED2 (Ub2) and UNBRANCHED3 (Ub3) [45], were shown to be associated with maize grain architecture. In soybean, a previous study reported that overexpression of *GmmiR156b* improved plant architecture, and consequently grain yield [21]. Ectopic overexpression of *GmSPL9d* reduced branch number in *Arabidopsis* [21]. However, the functions of GmSPL9d and its closest homologs, like GmSPL9a, GmSPL9b and GmSPL9c TFs (Additional file 1: Figure S1), in influencing soybean plant architecture remained to be determined.

Using the CRISPR/Cas9 system for genome editing, we were able to produce a number of single and higher-order mutants, particularly the quadruple mutant, for investigating the roles of these four TFs in forming soybean plant architecture. Specifically, we obtained the homozygous mutants *spl9b-1* and *spl9a*/*spl9b-1*/*spl9c*/*spl9d* after 4 generations (Additional file 2: Table S1). Detailed analyses of the mutants in different generations showed that the T4 higher-order mutants carrying various combinations of mutations exhibited various increased levels in node number on main stem, total node number per plant, branch number and dry weight compared with WT and *spl9b* single mutant plants (Fig. 4). Our results suggest that these four GmSPL9 TFs might very likely regulate these characteristics of plant architecture in soybean. All lower- and higher-order mutants in all combinations of these four *GmSPL9* genes should be obtained to clearly classify the important level of each of these four TFs in formation of soybean plant architecture. A new genome editing design is required to fulfill this task.

As discussed previously, we just obtained one *spl9b* single mutant plants with *Bar* negative, but did not obtained other three single mutants with individual mutation in *GmSPL9a*, *GmSPL9c* and *GmSPL9d* genes in order to clearly classify the functional roles of these four TFs. Thus, we could use only this stable *spl9b* single mutant as an example to analyze the expression patterns of all four *GmSPL9* genes. Our results showed that the *spl9b* single mutant plants had higher expression levels of all four *GmSPL9* genes in both SAM and leaves than WT (Fig. 5), while the transgenic plants overexpressing *GmSPL9b* displayed lower expression levels of *GmSPL9a*, *GmSPL9c* and *GmSPL9d* genes than WT plants (Additional file 7: Figure S5). This finding suggested that the GmSPL9b TF might negatively regulate the expression of *GmSPL9* genes in soybean, which might result in no or minor changes in the *spl9b* single mutant, when compared with WT plants, with respect to the examined phenotypic parameters (Fig. 4). Further studies need to be conducted using all combinations of single, double, triple and quadruple mutant plants of these four *GmSPL9* genes to examine their complex functions in soybean plant architecture and the feedback mechanism underlying the expression patterns of the examined *GmSPL9* genes. In addition, our data were obtained under artificial climate chamber conditions. Further studies under field conditions are required to reveal the roles of these four *GmSPL9* genes in regulating soybean plant architecture and especially grain yield, prior to using them in genetic engineering for improvement of soybean productivity.

## Conclusions

The CRISPR/Cas9 system currently has become a versatile tool to advance crop plant breeding. In our present study, we used CRISPR/Cas9-based multiple genome editing, and successfully obtained several mutants, including the quadruple mutant, for assessment of the functions of four closely homologous *GmSPL9* genes in formation of soybean plant architecture. Using these mutants, we found that the four *GmSPL9* genes may have redundant or independent roles in regulating soybean plant architecture, depending on the phenotypic trait(s) examined. Our data also suggested that the *GmSPL9b* gene can regulate the expression of the four *GmSPL9* genes, including itself, in soybean. Taken together, results of our studies improve the understanding of the application of CRISPR/Cas9 system and provide more knowledge on the regulation of plant architecture in soybean.

## Methods

### Plant materials and growth conditions

The soybean [*Glycine max* (L.) Merr.] cultivar Williams 82 was used for transformation. The WT plants, *GmmiR156b-*overexpressing transgenic plants (line #5 from Sun et al., 2018) [21], and mutant plants were cultivated in an artificial climate chamber under the conditions of 12-h light and 12-h dark photoperiod at 24 °C.

To investigate the plant architecture of transgenic plants, 17 T4-generation higher-order *spl9* mutant plants of different combinations (Fig. 4) were grown in an artificial climate chamber under the 15-h light/9-h dark photoperiodic conditions at 28 °C. The WT, *GmmiR156b-*overexpressing transgenic and the homozygous *spl9b-1* single mutant (*Bar* negative) plants were grown with 6 seedlings/each genotype (Fig. 4). The seeds of each genotype were germinated on moistened filter paper for 4 days at 28 °C and 60% humidity under 15-h light/9-h dark photoperiodic conditions. Germinated seedlings were transferred into 25 cm × 25 cm pot with each pot containing one seedling. All examined phenotypic parameters, including branch number (first branch number on main stem), node number on main stem and total node number per plant, were recorded at 50 DAE. To investigate the plastochron length in the T4 soybean mutants, the trifoliate leaves on main stem were recorded every 10 DAE from 20 to 50 DAE.

### Construction of phylogenetic tree

The full-length protein sequences of 43 soybean SPLs were retrieved from the Phytozome (www. Phytozome.net/) and used to construct a phylogenetic tree to study the relationships of the soybean SPLs with the AtSPL9, AtSPL15, OsSPL14 and OsSPL17 proteins, whose full-length protein sequences were also downloaded from Phytozome. MEGA v.7.0 was used to construct the Neighbour-Joining unrooted tree [46].

### Plasmid construction and soybean transformation

The nucleotide sequence of the four *GmSPL* genes were downloaded from Phytozome. The target sequence adaptors were designed using the web tool CRISPR-P (http://cbi.hzau.edu.cn/crispr/). The kanamycin resistance gene in the pYLCRISPR/Cas9P_35S_-B, which was received from Ma et al. [38], was replaced by the spectinomycin resistance *aadA* gene, resulting in the pYLCRISPR/Cas9P_35S_-BS. The four target sequence adaptors were integrated into different sgRNA expression cassettes and built into the pYLCRISPR/Cas9P_35S_-BS vector according to the protocol reported by Ma et al. (2015) [38]. Briefly, a digestion/ligation reaction for each sgRNA expression cassette was prepared as follows: 1 μL 10 × CutSmart buffer, 20 ng pYLsgRNA plasmid DNA, 0.5 μL target adapter, 3 U *Bsa*I-HF, 20 U T4 DNA ligase, 0.5 μL 10 × NEB T4 DNA ligase buffer and deionized H_2_O to a final volume of 10 μL. Subsequently, the digestion/ligation reaction was performed in a thermal cycler without using a heated lid at the following thermal cycling program: 10 cycles of 5 min at 37 °C and 5 min at 20 °C. Next, all four sgRNA cassettes were amplified by PCR using the products of digestion/ligation reaction as template and the site-specific primer pairs (Additional file 8: Table S2) at the following thermal cycling program: 22 cycles of 10 s at 95 °C, 15 s at 58 °C and 20 s at 68 °C. Equal amounts of the obtained PCR products were mixed and purified using a PCR product purification kit (Axygen, California, USA). Finally, a digestion/ligation reaction was prepared to assemble the four sgRNA cassettes into the pYLCRISPR/Cas9P_35S_-BS as follows: 1.5 μL 10 × CutSmart buffer, 100 ng pYLCRISPR/Cas9P_35S_-BS plasmid DNA, 100 ng pooled sgRNA cassettes (the mixture of four PCR products obtained from the previous step), 10 U *Bsa*I-HF, 40 U T4 DNA ligase, 0.5 μL 10 × NEB T4 DNA ligase buffer and deionized H_2_O to a final volume of 15 μL. Subsequently, the digestion/ligation reaction was performed in a thermal cycler without using a heated lid at the following thermal cycling program: 15 cycles of 5 min at 37 °C, 5 min at 10 °C and 5 min at 20 °C.

The obtained CRISPR/Cas9 plasmid carrying the sgRNA cassettes was transformed into *A. tumefaciens* strain EHA105, followed by the soybean transformation that was conducted according to the description previously reported by Cao et al., 2015 [37] with some modifications. Briefly, sterilized Williams 82 seeds were germinated in B5 medium for one day, and then the one-day-old germinated seedlings were vertically cut at cotyledonary node, and any remaining axial shoot/bud parts attached to the cotyledonary node were removed. Subsequently, the explants were wounded with a scalpel and dipped into the *A. tumefaciens* strain EHA105 carrying the CRISPR/Cas9 plasmid with the sgRNA cassettes built-in. After 4 days of co-cultivation in co-cultivation medium, the explants were transferred into the shoot induction medium without glufosinate. Seven days later, the explants were transferred into the shoot induction medium with 10 mg L^− 1^ glufosinate for 2 weeks. Subsequently, the explants were cultured in the shoot elongation medium containing 5 mg L^− 1^ glufosinate. When the elongated shoots were about 3 cm, they were transferred to rooting medium without further selection. Glufosinate (160 mg L^− 1^) was applied until the first trifoliate appeared to screen for T0, T1 and T2 transformants.

To obtain transgenic soybean plants overexpressing *GmSPL9b*, the *GmSPL9b*-pTF101 vector harboring the *GmSPL9b* gene under the *35S* promoter from cauliflower mosaic virus was used to transform the soybean cultivar Dongnong 50, according to the description previously reported by Cao et al., 2015 [37].

### DNA extraction and mutation screening

Genomic DNA was extracted from the leaves of each independent T0, T1, T2, T3 and T4 plant and used for PCR. The target site sequences were amplified by PCR with sequence-specific primer sets (Additional file 8: Table S2), and the PCR products were then separated by electrophoresis on 1.0% agarose in 1 × TAE buffer. The purified DNA fragments were sequenced and analyzed. The successfully edited types could be identified via sequence peaks and alignment to the reference sequences. The heterozygous mutants showed overlapping peaks near the target site, and the homozygous mutants were identified by sequence alignment with the WT sequence.

To screen the *spl9d* mutants in T2 generation, the seeds obtained from 10 T1-generation plants (four T1–10 plants and six T1–20 plants) were sown to sample DNAs from 120 independent T2 plants (12 independent T2 plants from each T1-generation plant). The DNAs of 12 independent T2 plants from each T1-generation plant were then pooled as a DNA template for PCR, resulting in a total of 10 DNA pools. To screen the *spl9d* mutants in T3 generation, similar protocol was conducted as described above in T2 generation. To screen the *spl9d* mutants in T4 generation, the DNA of every independent T4 plant was used as template for PCR. The PCR products were digested with *Eco*RI (New England Biolabs) and then separated by 1.0% agarose in 1 × TAE buffer.

### RNA isolation, cDNA synthesis and qRT-PCR analysis

The shoot apical meristem (SAM) and third fully developed trifoliate leaves from the bottom of the plants were sampled at 20th DAE and immediately frozen in liquid nitrogen. Total RNA was isolated from each sample using TRIzol reagent (Invitrogen, USA), and cDNA was synthesized for quantitative RT-PCR (qRT-PCR) to assess the transcript levels of *GmSPL9a* (*Glyma.02G177500*), *GmSPL9b* (*Glyma.09G113800*), *GmSPL9c* (*Glyma.03G143100*), *GmSPL9d* (*Glyma.19G146000*) and *GmTUB (Glyma.08G014200*) (as an internal control) as described previously in Cao et al., (2015) [37]. The primers used for qRT-PCR are listed in Additional file 8: Table S2. The qRT-PCR mixture was prepared by mixing 1 μL of the cDNA synthesis reaction mixture with 2.5 μL forward primer (final concentration 1.0 mM), 2.5 μL reverse primer (final concentration 1.0 mM), 10 μL of SYBR Premix Extaq Perfect Real Time (TAKARA Bio Inc., Japan) and water to a final volume of 20 μL. The qRT-PCR was performed using the program as essentially described in Nan et al. (2014) [47].

### Data analysis

Data of phenotype were analyzed with the SPSS (Version 21.0) using one-way analysis of variance. Data of the expression of genes were analyzed with the SPSS (Version 21.0) using the Student’s *t*-test analysis.

## Additional files

Additional file 1: **Figure S1.** Phylogenetic tree showing the relationships of 43 soybean SPLs with the AtSPL9, AtSPL15, OsSPL14 and OsSPL17 proteins. (DOCX 232 kb)

Additional file 2: **Table S1.** CRISPR/Cas9-meditated targeted mutagenesis of four *GmSPL9* genes in transgenic soybean plants of different generations. (DOCX 95 kb)

Additional file 3: **Figure S2.** Multiple alignment of the amino-acid sequences of (A) two edited types of *spl9b* mutants and (B) one edited type of *spl9c* mutant. (DOCX 426 kb)

Additional file 4: **Text S1.** The coding sequence (CDS) and protein sequence of *GmSPL9* genes, and mutated sequences obtained by CRISPR/Cas9-mediated mutagenesis. (DOCX 381 kb)

Additional file 5: **Figure S3.** Identification of ‘transgene-clean’ mutant lines. (DOCX 94 kb)

Additional file 6: **Figure S4.** The *spla9a/spl9b-1* double homozygous mutant plants showed slightly shorter plastochron length. (DOCX 165 kb)

Additional file 7: **Figure S5.** Effect of GmSPL9 transcription factor on expression of *GmSPL9a*, *GmSPL9c* and *GmSPL9d* in soybean leaves, as well as on soybean architecture. (DOCX 90 kb)

Additional file 8: **Table S2.** Primers used for PCR and qRT-PCR in this study. (DOCX 84 kb)

## Abbreviations

AM: Axillary meristem
AP2: Apetala 2
APC/C: Anaphase-promoting complex/cyclosome
Cas9: CRISPR-associated system 9
CRISPR: Clustered regularly interspaced short palindromic repeat
CTAB: Cetyltrimethyl ammonium bromide
DEP1: DENSE AND ERECT PANICLE 1
FT: Flower Locus T
GmILPA1: *Glycine max* INCREASED LEAF PETIOLE ANGLE 1
IPA1: IDEAL PLANT ARCHITECTURE 1
IPI1: IPA1 INTERACTING PROTEIN 1
QTLs: Quantitative trait loci
SAM: Shoot apical meristem
SBP: SQUAMOSA PROMOTER BINDING PROTEIN
SPL: SQUAMOSA PROMOTER BINDING PROTEIN-LIKE
TB1: TEOSINTE BRANCHED 1
TF: Transcription factor
TGA1: TEOSINTE GLUME ARCHITECTURE
TSH4: TASSELSHEATH4
Ub2: UNBRANCHED2
Ub3: UNBRANCHED3
WFP: WEALTHY FARMER’S PANICLE
WT: Wild-type
WUS: WUSCHEL
