# Supplementary figures and images for: CRISPR/Cas9-mediated targeted mutagenesis of GmSPL9 genes alters plant architecture in soybean
Source: BMC Plant Biol. 2019 Apr 8;19:131. doi: 10.1186/s12870-019-1746-6 (PMC6454688; doi:10.1186/s12870-019-1746-6)

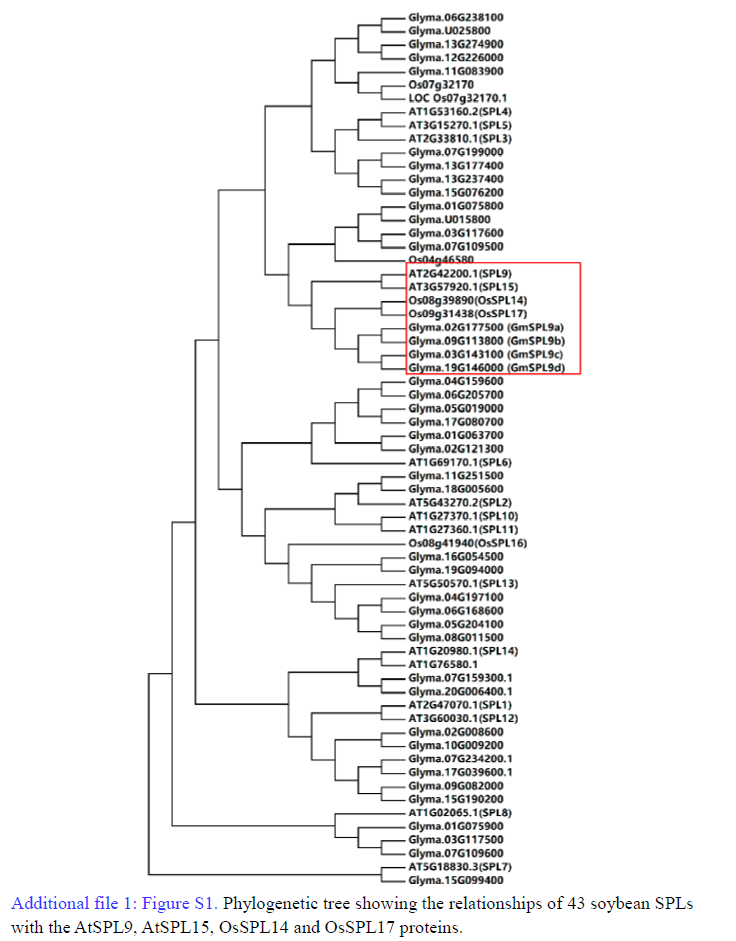

Supplement: Supplementary file 1 — Figure S1. Phylogenetic tree showing the relationships of 43 soybean SPLs with the AtSPL9, AtSPL15, OsSPL14 and OsSPL17 proteins. (DOCX 232 kb) [file 12870_2019_1746_MOESM1_ESM.docx]

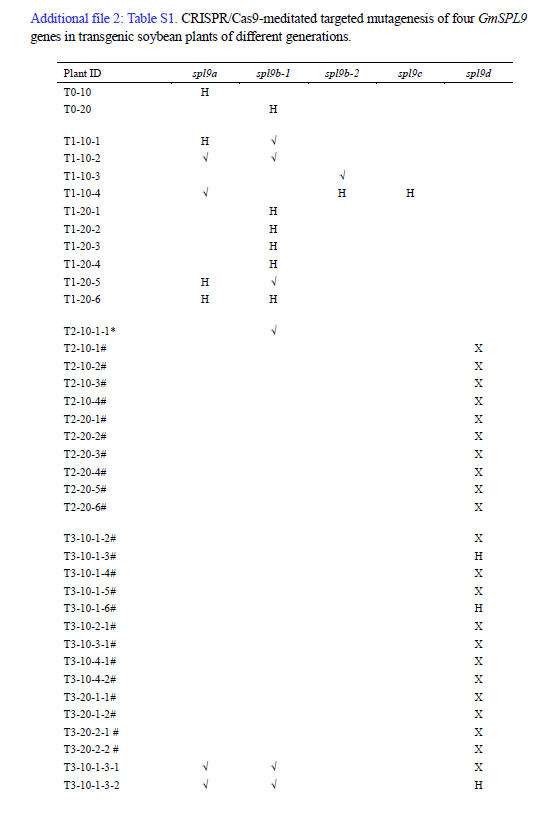


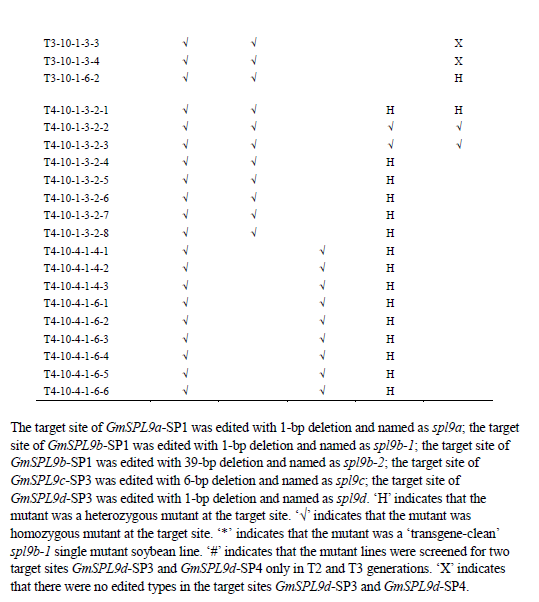

Supplement: Supplementary file 2 — Table S1. CRISPR/Cas9-meditated targeted mutagenesis of four GmSPL9 genes in transgenic soybean plants of different generations. (DOCX 95 kb) [file 12870_2019_1746_MOESM2_ESM.docx]

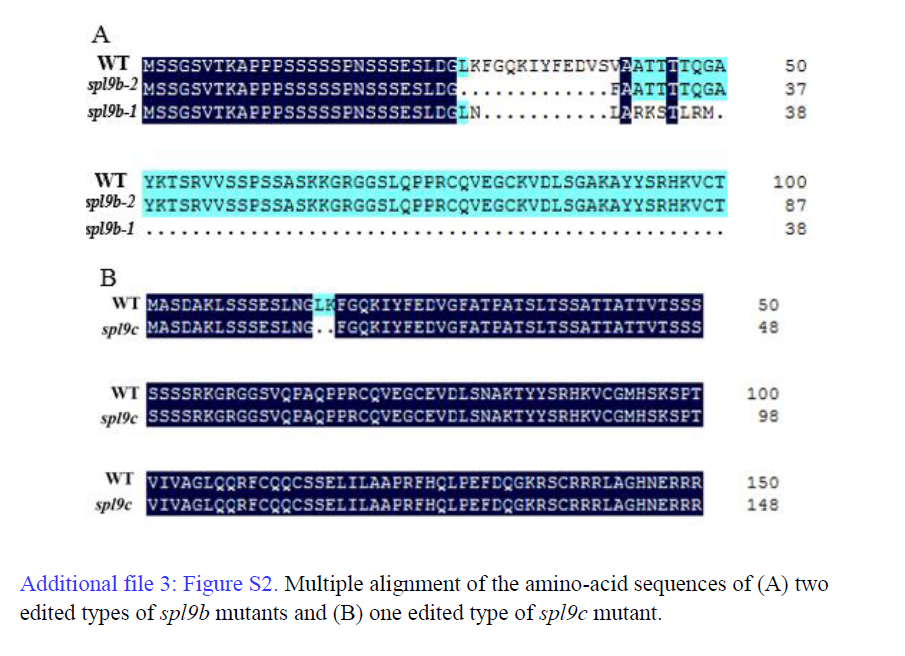

Supplement: Supplementary file 3 — Figure S2. Multiple alignment of the amino-acid sequences of (A) two edited types of spl9b mutants and (B) one edited type of spl9c mutant. (DOCX 426 kb) [file 12870_2019_1746_MOESM3_ESM.docx]

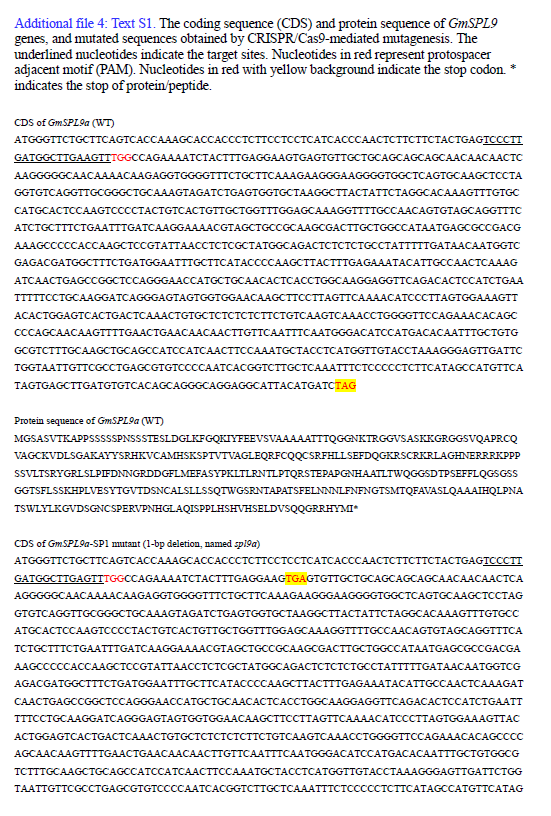


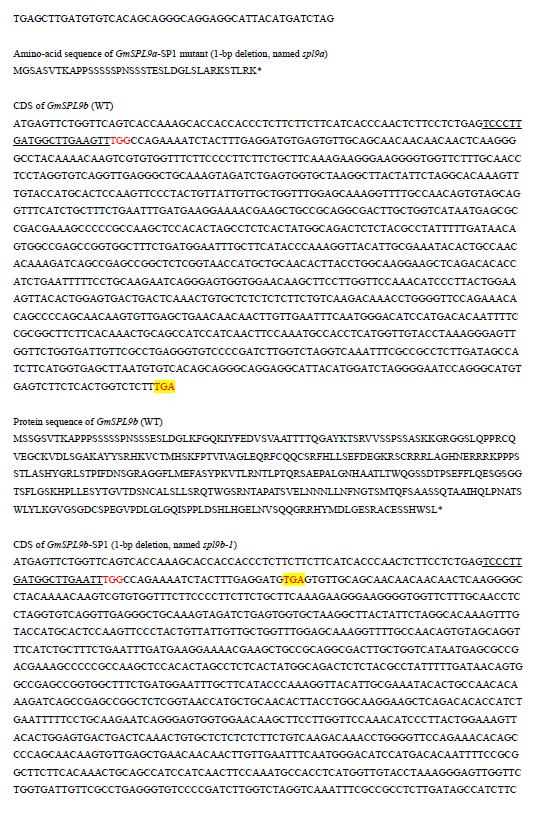


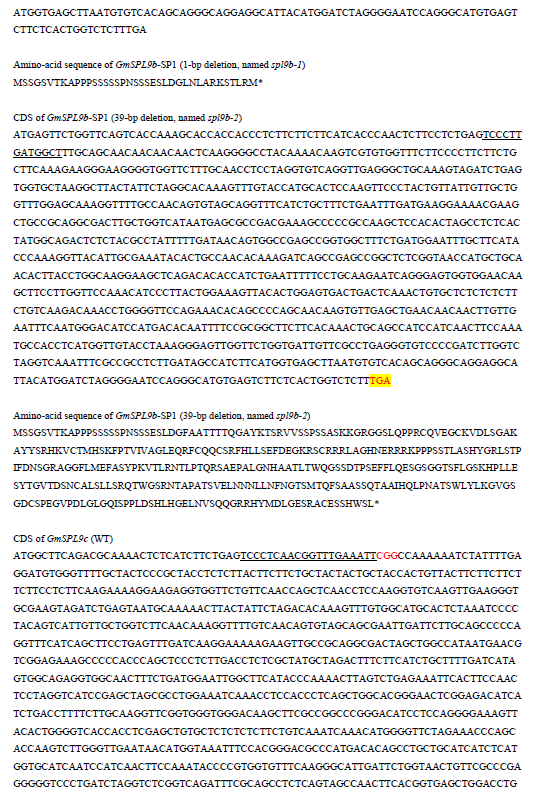


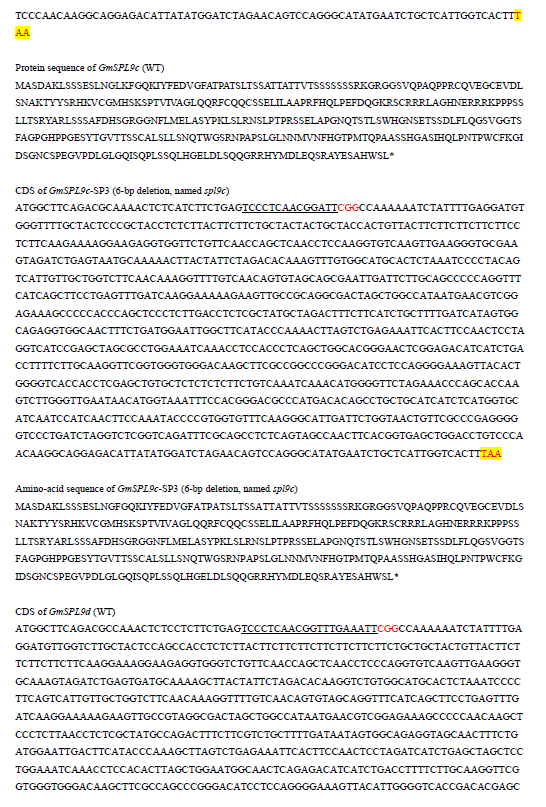


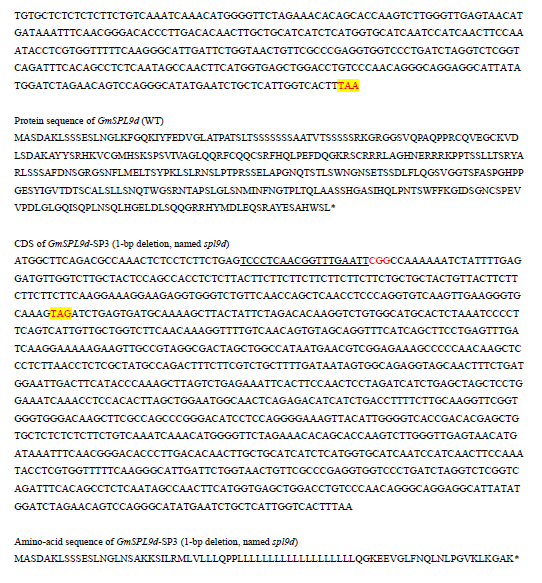

Supplement: Supplementary file 4 — Text S1. The coding sequence (CDS) and protein sequence of GmSPL9 genes, and mutated sequences obtained by CRISPR/Cas9-mediated mutagenesis. (DOCX 381 kb) [file 12870_2019_1746_MOESM4_ESM.docx]

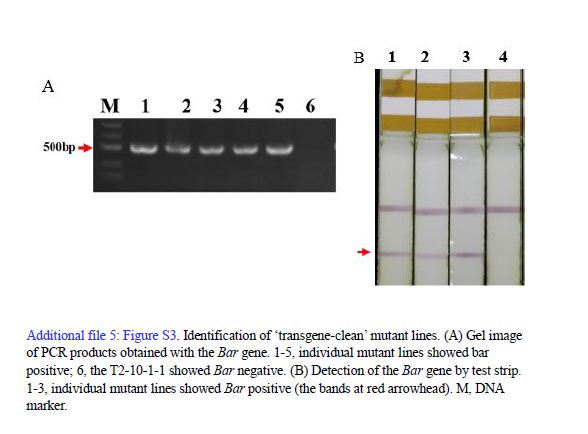

Supplement: Supplementary file 5 — Figure S3. Identification of ‘transgene-clean’ mutant lines. (DOCX 94 kb) [file 12870_2019_1746_MOESM5_ESM.docx]

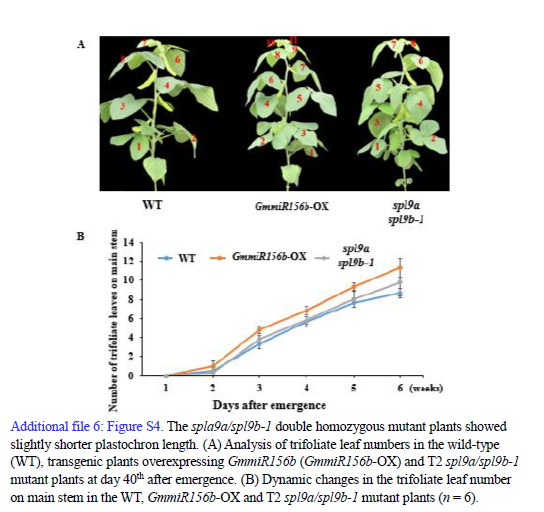

Supplement: Supplementary file 6 — Figure S4. The spla9a/spl9b-1 double homozygous mutant plants showed slightly shorter plastochron length. (DOCX 165 kb) [file 12870_2019_1746_MOESM6_ESM.docx]

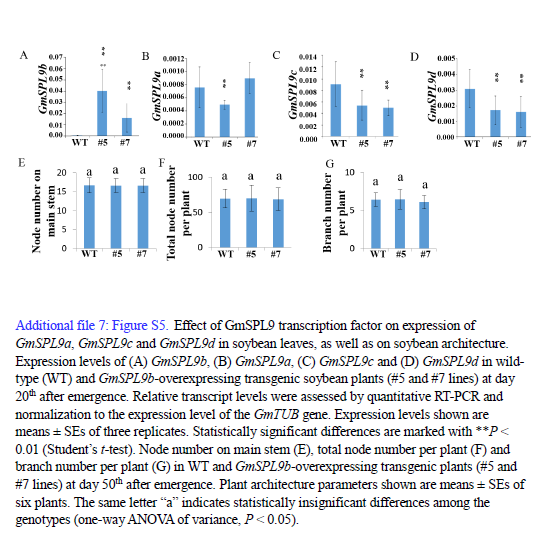

Supplement: Supplementary file 7 — Figure S5. Effect of GmSPL9 transcription factor on expression of GmSPL9a, GmSPL9c and GmSPL9d in soybean leaves, as well as on soybean architecture. (DOCX 90 kb) [file 12870_2019_1746_MOESM7_ESM.docx]

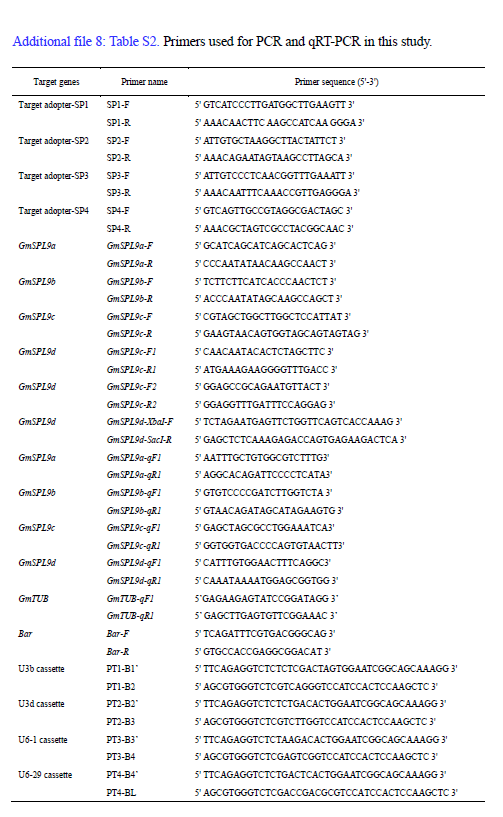

Supplement: Supplementary file 8 — Table S2. Primers used for PCR and qRT-PCR in this study. (DOCX 84 kb) [file 12870_2019_1746_MOESM8_ESM.docx]
